# Recombinant human proteoglycan-4 reduces phagocytosis of urate crystals and downstream nuclear factor kappa B and inflammasome activation and production of cytokines and chemokines in human and murine macrophages

**DOI:** 10.1186/s13075-018-1693-x

**Published:** 2018-08-29

**Authors:** Marwa Qadri, Gregory D. Jay, Ling X. Zhang, Wendy Wong, Anthony M. Reginato, Changqi Sun, Tannin A. Schmidt, Khaled A. Elsaid

**Affiliations:** 1 Department of Biomedical and Pharmaceutical Sciences, Chapman University School of Pharmacy, Rinker Health Sciences Campus, 9401 Jeronimo Road, Irvine, CA 92618 USA; 2 Department of Emergency Medicine, Rhode Island Hospital, Providence, RI USA; 3 Department of Biomedical Engineering, Brown University, Providence, RI USA; 4 Division of Rheumatology and Department of Dermatology, Rhode Island Hospital, Providence, RI USA; 5 Biomedical Engineering Department, School of Dental Medicine, University of Connecticut Health Center, Farmington, CT USA

**Keywords:** Gout, Proteoglycan-4, Macrophages, Lubricin, Urate, CD44, TLR2, TLR4

## Abstract

**Background:**

Gout is an inflammatory arthritis caused by monosodium urate monohydrate (MSU) crystals’ joint deposition. MSU phagocytosis by resident macrophages is a key step in gout pathogenesis. MSU phagocytosis triggers nuclear factor kappa B (NFκB) activation and production of cytokines and chemokines. Proteoglycan-4 (PRG4) is a glycoprotein produced by synovial fibroblasts and exerts an anti-inflammatory effect in the joint mediated by its interaction with cell surface receptor CD44. PRG4 also binds and antagonizes TLR2 and TLR4. The objective of this study is to evaluate the efficacy of recombinant human PRG4 (rhPRG4) in suppressing MSU-induced inflammation and mechanical allodynia in vitro and in vivo.

**Methods:**

THP-1 macrophages were incubated with MSU crystals ± rhPRG4 or bovine submaxillary mucin (BSM), and crystal phagocytosis, cytokines and chemokines expression and production were determined. NFκB p65 subunit nuclear translocation, NLRP3 induction, caspase-1 activation and conversion of proIL-1β to mature IL-1β were studied. MSU phagocytosis by *Prg4*^*+/+*^ and *Prg4*^*−/−*^ peritoneal macrophages was determined in the absence or presence of rhPRG4, BSM, anti-CD44, anti-TLR2, anti-TLR4 and isotype control antibodies. Rhodamine-labeled rhPRG4 was incubated with murine macrophages and receptor colocalization studies were performed. Lewis rats underwent intra-articular injection of MSU crystals followed by intra-articular treatment with PBS or rhPRG4. Weight bearing and SF myeloperoxidase activities were determined.

**Results:**

rhPRG4 reduced MSU crystal phagocytosis at 4 h (*p < 0.01*) and IL-1β, TNF-α, IL-8 and MCP-1 expression and production at 6 h (*p < 0.05*). BSM did not alter MSU phagocytosis or IL-1β production in human and murine macrophages. rhPRG4 treatment reduced NFκB nuclear translocation, NLRP3 expression, caspase-1 activation and generation of mature IL-1β (*p < 0.05*). MSU-stimulated IL-1β production was higher in *Prg4*^*−/−*^ macrophages compared to *Prg4*^*+/+*^ macrophages (*p < 0.001*). rhPRG4, anti-CD44, anti-TLR2 and anti-TLR4 antibody treatments reduced MSU phagocytosis and IL-1β production in murine macrophages (*p < 0.05*). rhPRG4 preferentially colocalized with CD44 on *Prg4*^*−/−*^ peritoneal macrophages compared to TLR2 or TLR4 (*p < 0.01*). rhPRG4 normalized weight bearing and reduced SF myeloperoxidase activity compared to PBS in vivo.

**Conclusion:**

rhPRG4 inhibits MSU crystal phagocytosis and exhibits an anti-inflammatory and anti-nociceptive activity in vitro and in vivo. rhPRG4’s anti-inflammatory mechanism may be due to targeting CD44 on macrophages.

## Background

Gout is an inflammatory arthritis characterized by deposition of monosodium urate monohydrate (MSU) crystals in synovial joints and periarticular tissues [1, 2]. Gout is characterized by painful episodes of intermittent acute monoarthritis, most often in peripheral joints such as the first metatarsophalangeal and knee joints, in the midst of asymptomatic periods [2, 3]. Tissue MSU crystal deposits initiate inflammation in resident macrophages, mediated in part by pattern recognition receptors of the innate immune system, such as toll-like receptors (TLR2 and TLR4) [4–8]. Other endogenous TLR ligands, such as myeloid-related proteins 8 and 14 and long chain fatty acids may play a role in priming macrophages to the inflammatory effect of MSU crystals [9, 10]. Priming macrophages stimulates nuclear factor kappa B (NFκB) nuclear translocation, proIL-1β expression and induces the expression of NACHT, LRR and PYD-containing protein 3 (NLRP3) inflammasome components: NLRP3 protein, ASC adaptor protein, and caspase-1 [11–13]. NFκB translocation results in inducing the expression and secretion of proinflammatory cytokines, e.g. interleukin-1 beta (IL-1β) and tumor necrosis factor alpha (TNF-α) and chemokines, e.g. interleukin-8 (IL-8) and monocyte chemoattractant protein-1 (MCP-1) [14–17]. Particulate danger signals e.g. MSU crystals are thought to cause lysosomal disruption following their endocytosis by macrophages and thus trigger inflammasome activation and conversion of proIL-1β to IL-1β with downstream enhancement of the inflammatory cascade and inflammatory cell influx to the affected joint [18–22].

Lubricin/Proteoglycan-4 (PRG4) is a mucinous glycoprotein secreted by synovial fibroblasts and superficial zone articular chondrocytes [23–25]. PRG4 is the major lubricating constituent of synovial fluid (SF) and a biological role for PRG4 has been described. The recombinant form of PRG4 exhibits an anti-inflammatory role characterized by its ability to compete with hyaluronan on binding to the CD44 receptor [26]. The downstream effect of engaging CD44 by PRG4 is the inhibition of IL-1β and TNF-α induced NFκB nuclear translocation in synoviocytes from patients with RA and OA [26, 27]. The autocrine anti-inflammatory role of PRG4 on synovial fibroblasts was shown to be mediated by its ability to inhibit the degradation of cytosolic inhibitor kappa B alpha (IκB-α) in a CD44-dependent manner [27]. Related to TLRs, recombinant human PRG4 (rhPRG4) binds to, and regulates agonist-induced activation of TLR2 and TLR4 [28, 29]. Supplementation of OA and RA synovial fluid aspirates with the native form of PRG4 inhibited TLR2 and TLR4 activation by these aspirates [29].

The objective of this study is to evaluate the efficacy of rhPRG4 related to modulation of MSU crystal uptake by human and murine macrophages and subsequent cellular activation and induction of proinflammatory cytokines and chemokines expression and production. Furthermore, we studied the ability of rhPRG4 to reduce inflammation and acute mechanical allodynia in a rat model of intra-articular MSU challenge. We hypothesized that rhPRG4 inhibits MSU crystal phagocytosis by macrophages through the inhibition of TLR receptors or CD44, resulting in a significant reduction in IL-1β, TNF-α, IL-8 and MCP-1 expression and production and an anti-inflammatory and an anti-nociceptive effect in vivo.

## Methods

### Differentiation of THP-1 monocytes into macrophages and studies of time-dependent MSU phagocytosis and impact of rhPRG4 treatment

THP-1 monocytes (ATCC, USA) were cultured to a density of 1.5 × 10^6^ cells/ml in 75 cm^2^ flask in RPMI 1640 medium supplemented with 10% heat-inactivated fetal bovine serum (FBS), 10 mM HEPES, 2 mM glutamine, 100 U/L Penicillin and 100μg/ml streptomycin and maintained at 37 °C. In sterile 12 well plates (Corning, Sigma Aldrich, USA), 500,000 cells in 2 ml RPMI 1640 media were differentiated into macrophages by incubation with phorbol 12-myristate-13-acetate (PMA; Sigma Aldrich) to a final concentration of 5 ng/ml for 48 h [30]. Subsequently, media supernatants were removed and wells were washed with sterile PBS to remove any unattached cells and new RPMI 1640 media was added.

THP-1 macrophages were treated with endotoxin-free MSU crystals (100μg/ml; Invivogen, USA) ± bovine submaxillary mucin (BSM; molecular mass > 1000 KDa) (Sigma Aldrich) (25 μg/ml) or rhPRG4 (molecular mass is approximately 240 KDa) (100 μg/ml) for 2 and 4 h at 37 °C. rhPRG4 is an endotoxin-free full-length product produced by CHO-M cells (Lubris, Framingham, MA, USA) [31]. Subsequently, adherent macrophages were harvested via trypsinization, pelleted and washed with PBS. The phagocytosis of MSU crystals was determined by analyzing change in cell side-scatter using a flow cytometer (BD FACSVerse, BD Biosciences, USA). Two regions of interest were identified. P1 represents the THP-1 macrophage population in the absence of MSU exposure. P2 represents the THP-1 macrophage population with increased side-scatter indicative of MSU internalization. Data are presented as the mean percentage of cells in the P2 region across different time points and different treatments, and were derived from four independent experiments with duplicate wells per group. All flow cytometry experiments were performed using the same acquisition parameters.

### Comparative effect of rhPRG4 and BSM on MSU-stimulated production of proinflammatory cytokines and chemokines

THP-1 macrophages (500,000 cells per well) were treated with MSU (100μg/ml) ± rhPRG4 (100μg/ml) or BSM (25μg/ml) for 6 h followed by collection of media supernatants. Media concentrations of IL-1β, TNF-α, IL-8 and MCP-1 were determined using ELISA kits (R&D Systems, USA). Data represent the mean ± S.D. of four independent experiments with duplicate wells per group.

### Nuclear p65 NFκB translocation and NLRP3 inflammasome activation following MSU challenge and impact of rhPRG4 treatment

NFκB p65 subunit translocation studies were performed as previously described [32]. THP-1 macrophages (600,000 cells per well) were treated with MSU (100μg/ml) ± rhPRG4 (50 and 100μg/ml) for 1 h followed by cell harvest and nuclear protein extraction. Nuclear levels of p65 subunit were determined using a DNA binding ELISA assay (Abcam) and were normalized to total nuclear protein content using the micro BCA assay and expressed as detectable NFκB p65 levels normalized to untreated controls. Data represent the mean ± S.D. of three independent experiments with duplicate wells per treatment.

THP-1 macrophages (500,000 cells per well) were treated with MSU (100μg/ml) ± rhPRG4 (100 and 200μg/ml) for 12 h. A positive control treatment (H_2_O_2_; 5 mM) was also included in the absence or presence of rhPRG4 (200μg/ml). Subsequently, cells were washed twice with ice-cold PBS and lysed on ice in RIPA buffer for 30 min and centrifuged for 5 min (16,000 g at 4 °C). The supernatant was collected and total cellular protein was quantified using BCA protein assay kit (ThermoFisher Scientific). Equal amounts of protein (40–50 μg) were loaded and separated by a 12% SDS-PAGE gel. Following blotting, membranes were blocked using 5% non-fat dry milk and probed with primary antibodies overnight at 4 °C. These antibodies included proIL-1β (D3U3E; Cell Signal Technology, USA), cleaved IL-1β (D3A3Z; Cell Signal Technology), NLRP3 (D2P5E; Cell Signal Technology), and capase-1 (MAB6216; R&D Systems). Target proteins were detected using IRDye secondary goat anti-mouse or goat anti-rabbit antibodies (LI-COR Biosciences, USA) and visualized with LI-COR Odyssey Blot Imager (LI-COR Biosciences).

### Dose-dependent effect of rhPRG4 treatment on MSU-induced proinflammatory cytokines and chemokines gene expression and production in THP-1 macrophages

THP-1 macrophages (500,000 cells per well) were treated with MSU (100μg/ml) ± rhPRG4 (25, 50, 100 and 200μg/ml) for 6 h. Total RNA was extracted using TRIzol reagent (Thermo Fisher Scientific), and RNA concentrations were determined with a NanoDrop ND-2000 spectrophotometer (NanoDrop Technologies, USA). cDNA was synthesized using Transcriptor First Strand cDNA Synthesis Kit (Roche, USA). Quantitative PCR (qPCR) was performed on Applied Biosystems Step One Plus Real-Time PCR System (Thermo Fisher Scientific) using TaqMan Fast Advanced Master Mix (Life Technologies, USA). Genes of interest included IL-1β (Hs00174097_m1, ThermoFisher Scientific), TNF-α (Hs01113624_g1, ThermoFisher Scientific), IL-8 (Hs00174103_m1, ThermoFisher Scientific) and MCP-1 (Hs00234140_m1, ThermoFisher Scientific). The cycle threshold (Ct) value of target genes was normalized to the Ct value of GAPDH (Hs02758991_g1; Thermo Fisher Scientific) in the same sample, and the relative expression was calculated using the 2^-ΔΔCt^ method [33]. Data are presented as fold target gene expression compared to untreated control. Data represent mean ± S.D. of three independent experiments with duplicate wells per treatment.

THP-1 macrophages (500,000 cells per well) were treated with MSU (100μg/ml) ± rhPRG4 (100 and 200μg/ml) for 24 H*. media* concentrations of IL-1β, TNF-α, IL-8 and MCP-1 were determined using commercially available ELISA kits (R&D Systems). Data represent the mean ± S.D. of three independent experiments with duplicate wells per group.

### Isolation of peritoneal macrophages from *Prg4*^*+/+*^ and *Prg4*^*−/−*^ mice, phagocytosis of MSU crystals by murine macrophages and downstream production of IL-1β and comparative efficacy of rhPRG4, anti-CD44, anti-TLR2 and anti-TLR4 antibody treatments

The phenotype of the *Prg4*^*−/−*^ mouse has been previously reported [34], and is characterized by cartilage degeneration and a hyperplastic synovium contributing to joint failure [34]. The *Prg4*^*−/−*^ and *Prg4*^*+/+*^ mouse colonies are maintained at Rhode Island Hospital. *Prg4*^*−/−*^ mouse is also commercially available (stock #025737; The Jackson Laboratory, Maine, USA). Isolation of murine peritoneal macrophages was performed as previously described [35] following IACUC approval at Rhode Island Hospital. A total of 20 *Prg4*^*+/+*^ and 20 *Prg4*^*−/−*^ mice were euthanized. Subsequently, the abdomen of each mouse was soaked with 70% alcohol and a small incision was made along the midline with scissors. Using blunt dissection, the abdominal skin was retracted to expose the intact peritoneal wall. A 27 G needle attached to a 10 ml syringe filled with sterile cold PBS was inserted through the peritoneal wall at the midline and injected into each mouse, aspirated slowly from the peritoneum, and peritoneal macrophages cells were collected. Subsequently, cells were centrifuged at 10,000 rpm and 4 °C for 10 min. Pelleted cells were re-suspended in RPMI 1640 medium supplemented with 10% FBS and 1% Penicillin/Streptomycin.

Murine peritoneal macrophages were plated onto sterile chamber slides (ThermoFisher Scientific) at a concentration of 1.3 × 10^6^ cells/well. Cells were allowed to adhere by incubation at 37 °C for 24 h. Following incubation, media and non-adherent cells were removed and fresh media was added. Treatments included untreated control cells, MSU (100μg/ml) ± rhPRG4 (100μg/ml), BSM (25μg/ml), anti-CD44 (Abcam; 2μg/ml), anti-TLR2 (Abcam; 2μg/ml), anti-TLR4 (Abcam; 2μg/ml) and isotype control (IC; 2μg/ml) (Abcam) antibodies. Incubations were performed for 4 and 24 h. Subsequently, slides were washed once with PBS and then fixed with 4% formalin for 15 min. Slides were subsequently washed with PBS and cells were permeabilized with 0.1% Triton X100 for 10 min. After washing with PBS for three times, slides were mounted with DAPI mounting medium (Vector Lab, USA) and viewed under a microscope (Nikon E800). The number of intracellular MSU crystals in 8 areas for a total of 900 cells was determined and the total number of MSU crystals was reported. Data represent the mean ± S.D. of four to five independent experiments. Media supernatants were assayed for IL-1β concentrations using a murine ELISA kit (R&D Systems).

### Colocalization of rhPRG4 and CD44, TLR2 and TLR4 receptors in *Prg4*^*−/−*^ peritoneal macrophages

Isolation and culture of *Prg4*^*−/−*^ peritoneal macrophages was performed as described above. Rhodamine labeling of rhPRG4 was performed using the Pierce NHS-Rhodamine Antibody Labeling Kit (Thermo Fisher Scientific). Rhodamine labeled rhPRG4 (25μg/ml) was incubated with *Prg4*^*−/−*^ macrophages for 2 h. Subsequently, media was removed and cells were washed with PBS and fixed using 4% formalin for 15 min at room temperature. Cells were then permeabilized with 0.2% Triton X-100 for 10 min and subsequently blocked with 2% BSA for 30 min. Cells were incubated with CD44 antibody, TLR2 antibody, TLR4 antibody or an isotype control (Abcam) (1:200 dilution) overnight at 4 °C. Cells were then washed with PBS and incubated with Alexa Fluor 488 goat anti-rabbit IgG (Thermo Fisher Scientific) at 1:200 dilution for 1 h at room temperature. After washing with PBS for three times, slides were mounted with DAPI mounting medium (Vector Lab). Confocal images were acquired with a Nikon C1si confocal microscope (Nikon Inc., USA) using diode lasers 402, 488 and 561. Serial optical sections were obtained sequentially with EZ-C1 computer software’s frame lambda setting. Z series sections were collected at 0.2 μm with a 60× Plan Apo, 1.4 numerical aperture lens. Six to seven fields were collected per sample for a total minimum number of 100 cells. All colocalization analyses were performed on deconvolved, 3D acquisitions (Elements version 3.2, Nikon Inc.). In each Z stack, cells were outlined and analyzed with Nikon’s colocalization macro. Pearson’s Correlation Coefficient was used to determine colocalization. A minimum threshold of Pearson’s Coefficient > 0.5 was used to indicate positive colocalization. Data is presented as percent of cells positive for rhPRG4 and receptor colocalization and is expressed as mean ± standard deviation of 3 experiments.

### Crystal-induced inflammation and mechanical allodynia in the rat and the impact of rhPRG4 treatment

Male Lewis rats (*n* = 40; 10 weeks old) (Charles River, USA) were randomly assigned to three experimental groups; MSU only, MSU + PBS or MSU + rhPRG4. All animals received an intra-articular injection of pyrogen-free MSU suspension (50 μl; 5 mg/ml). Intra-articular injections were performed under gas anesthesia (5% isoflurane). Intra-articular injections were performed in the right knee joints. The skin around the right knee joint was shaved and the injection site was cleansed using a topical iodine-based antiseptic and 70% isopropranolol. At 1 h following MSU injection, animals received PBS (50 μl), rhPRG4 (50 μl; 1 mg/ml) or remained untreated. We have also included 4 animals that received intra-articular PBS (50 μl).

Static weight bearing of the hind limbs of animals at baseline and at 3 and 6 h post-MSU injection (*n* = 12 in the MSU alone group and *n* = 14 in PBS and rhPRG4-treated animals) and at 24 h post-MSU injection (*n* = 5 in MSU alone and *n* = 7 in PBS and rhPRG4-treated animals) was measured using an Incapacitance Meter (Harvard Apparatus, USA). Data are presented as differential weight bearing between the hind right limb and the hind left limb. Animals were euthanized either at 6 h (*n* = 7 in each group) or at 24 h (*n* = 5 in MSU alone and *n* = 7 in PBS and rhPRG4-treated animals) following MSU challenge. Lavaging of the right knee joint was performed by injecting 100 μl of normal saline followed by joint flexion and extension and aspirating ~ 20–30 μl of SF lavage. Animal sera were also collected. Myeloperoxidase (MPO) activity in SF lavage samples was measured using a commercially available kit (Abcam). SF lavage and serum urea concentrations of each animal were determined using a urea assay kit (Abcam) and the fold dilution in SF lavage was calculated [36, 37]. Data are expressed as MPO activity (μU) adjusted to fold urea dilution. SF lavaging was performed for 2 untreated control animals and 4 PBS-injected animals and SF lavage MPO activity was determined as described above. All animal studies were approved by the IACUC committee at MCPHS University.

### Statistical analyses

Statistical analyses of gene expression data were performed using ΔCt values (C_t_ target gene-C_t_ GAPDH) for each gene of interest in each experimental group and data were graphically presented as fold expression relative to untreated controls using the 2^-ΔΔCt^ method. Continuous variables were initially evaluated whether they satisfy the requirements for parametric statistical tests. Statistical significance comparing two groups or multiple groups with parametric data was assessed by Student’s *t* test or ANOVA followed by post-hoc multiple comparisons using Tukey’s post-hoc test. Statistical significance comparing two groups or multiple groups with nonparametric data was assessed by Rank Sum test or ANOVA on the ranks. Analysis of MSU phagocytosis by THP-1 macrophages following 2 and 4-h incubations and impact of rhPRG4 or BSM treatments was performed using 2-way ANOVA. A *p* value of < 0.05 was considered statistically significant.

## Results

### rhPRG4 treatment reduced MSU crystal phagocytosis by THP-1 macrophages

**Fig. 1:**
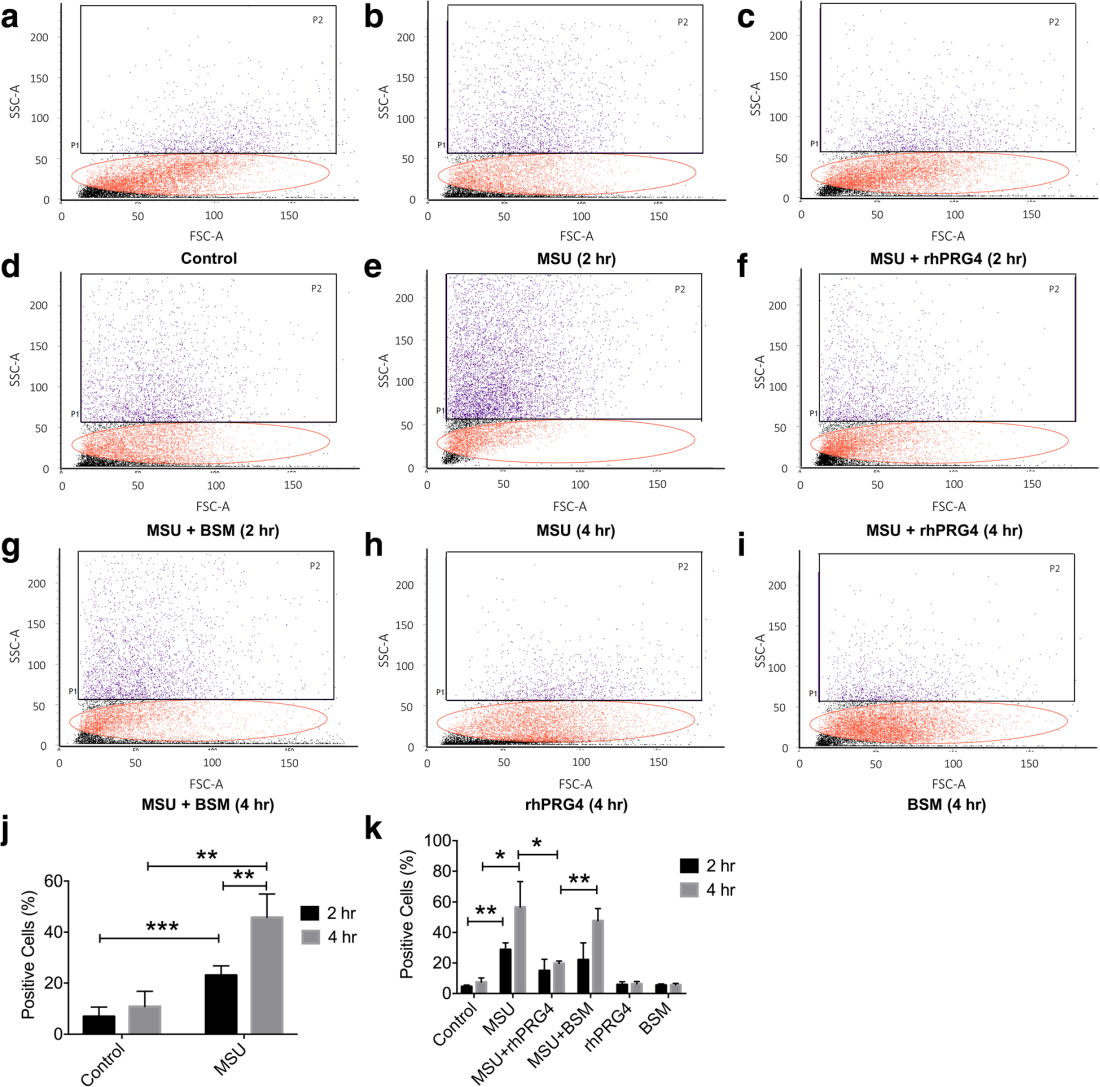
Time and treatment-dependent phagocytosis of monosodium urate monohydrate (MSU) crystals by differentiated human THP-1 macrophages using flow cytometry and impact of recombinant human proteoglycan-4 (rhPRG4) or bovine submaxillary mucin (BSM) treatments following 2 and 4-h incubations. Quantitative determination of MSU phagocytosis was performed using the percentage of cells in the P2 region of interest. Data represent the mean ± S.D. of four independent experiments. *p < 0.001; **p < 0.01; ***p < 0.05. **a** Representative flow cytometry scatterplot of untreated human THP-1 macrophages. **b** Representative flow cytometry scatterplot of MSU-treated THP-1 macrophages for 2 h. **c** Representative flow cytometry scatterplot of MSU + rhPRG4 (100 μg/ml)-treated THP-1 macrophages for 2 h. **d** Representative flow cytometry scatterplot of MSU + BSM (25 μg/ml)-treated THP-1 macrophages for 2 h. **e** Representative flow cytometry scatterplot of MSU-treated THP-1 macrophages for 4 h. **f** Representative flow cytometry scatterplot of MSU + rhPRG4 (100 μg/ml)-treated THP-1 macrophages for 4 h. **g** Representative flow cytometry scatterplot of MSU + BSM (25 μg/ml)-treated THP-1 macrophages for 4 h. **h** Representative flow cytometry scatterplot of rhPRG4 (100 μg/ml)-treated THP-1 macrophages for 4 h. **i** Representative flow cytometry scatterplot of BSM (25 μg/ml)-treated THP-1 macrophages for 4 h. **j** Phagocytosis of MSU crystals by THP-1 macrophages was higher in following 4-h incubation compared to 2-h incubation. **k** rhPRG4 treatment reduced MSU phagocytosis by THP-1 macrophages at 4 h compared to BSM

Representative MSU phagocytosis flow cytometry scatterplots are presented in Fig. 1. Qualitatively, THP-1 macrophages internalized MSU crystals at 2 and 4 h with more cells appearing in the P2 region of interest at 4 h compared to 2 h (Fig. 1b and e). rhPRG4 treatment appeared to reduce the number of THP-1 macrophages in the P2 region, especially following incubation for 4 h (Fig. 1f). BSM treatment as a negative control mucin did not appear to modify MSU phagocytosis by THP-1 macrophages (Fig. 1d and g). rhPRG4 or BSM alone did not appear to alter the side scattering of THP-1 macrophages (Fig. 1h and i). The percentage of positive cells in the 2-h MSU group was higher than the percentage of positive cells in the corresponding control group (*p* = 0.022) (Fig. 1j). Similarly, the percentage of positive cells in the 4-h MSU group was higher than the percentage of positive cells in the corresponding control group (*p* = 0.0002). MSU phagocytosis by THP-1 macrophages at 4 h was higher than MSU phagocytosis by THP-1 macrophages at 2 h (*p* = 0.003). rhPRG4 or BSM treatments did not alter MSU phagocytosis by THP-1 macrophages at 2 h (*p* = 0.461; *p* = 0.999) (Fig. 1k). In contrast, rhPRG4 significantly reduced MSU phagocytosis by THP-1 macrophages at 4 h (*p* < 0.001). BSM treatment did not alter MSU phagocytosis by THP-1 macrophages at 4 h (*p* = 0.981), and the percentage of positive cells in the MSU + BSM group was higher than the percentage of positive cells in the MSU + rhPRG4 group (*p* = 0.001). There was no significant difference in percentage of positive cells between rhPRG4-treated or BSM-treated macrophages and untreated controls at 2 or 4 h (*p* > 0.999 for all comparisons).

### rhPRG4 treatment reduced MSU stimulated cytokine and chemokine gene expression and production mediated by a reduction in NLRP3 inflammasome activation and NFκB nuclear translocation

**Fig. 2:**
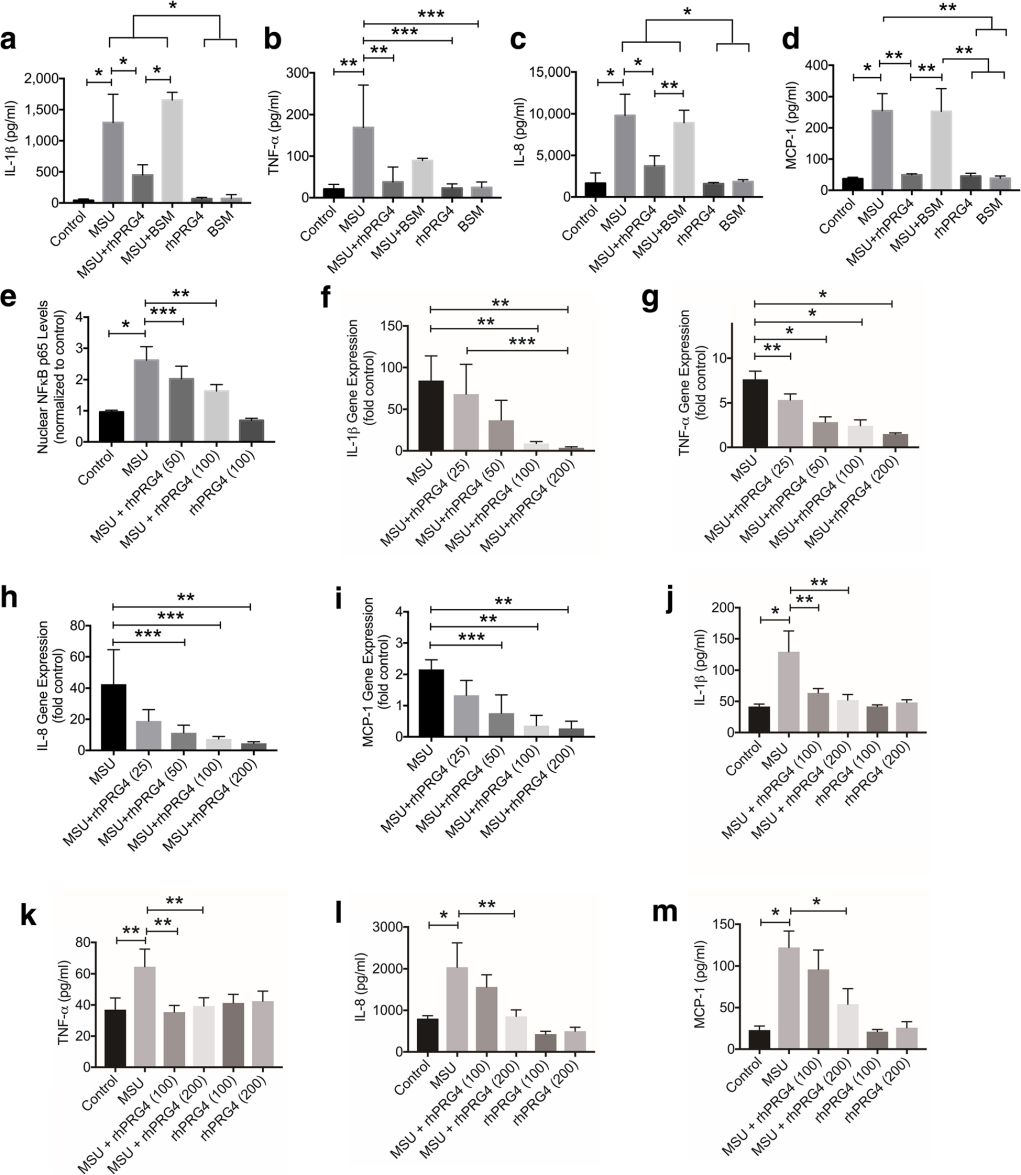
Impact of recombinant human proteoglycan-4 (rhPRG4) treatment on monosodium urate monohydrate (MSU) crystal-induced expression and production of proinflammatory cytokines and chemokines and nuclear factor kappa b (NFκB) p65 subunit nuclear translocation in THP-1 macrophages. Cytokines included interleukin-1 beta (IL-1β) and tumor necrosis factor alpha (TNF-α). Chemokines included interleukin-8 (IL-8) and monocyte chemoattractant protein-1 (MCP-1). Gene expression data are presented as fold induction of proinflammatory cytokines and chemokines gene expression compared to control untreated THP-1 macrophages. THP-1 macrophages were treated with MSU crystals (100μg/ml) ± rhPRG4 (100μg/ml) or bovine submaxillary mucin (BSM; 25μg/ml) for 6 h (**a** through **d**). NFκB p65 subunit nuclear translocation in THP-1 macrophages was performed at 1 h following MSU challenge (100μg/ml). Gene expression studies were performed at 6 h (**f** through **i**) and cytokine and chemokine media concentrations were determined at 24 h (**j** through **m**). Data represent the mean ± S.D. of three to four independent experiments with duplicate wells per group. *p < 0.001; **p < 0.01; ***p < 0.05. **a** rhPRG4 treatment reduced MSU-stimulated production of IL-1β by THP-1 macrophages. **b** rhPRG4 treatment reduced MSU-stimulated production of TNF-α by THP-1 macrophages. **c** rhPRG4 treatment reduced MSU-stimulated production of IL-8 by THP-1 macrophages. **d** rhPRG4 treatment reduced MSU-stimulated production of MCP-1 by THP-1 macrophages. **e** rhPRG4 treatment reduced MSU-stimulated NFκB p65 subunit nuclear translocation in THP-1 macrophages. **f** rhPRG4 (100 and 200μg/ml) treatment reduced IL-1β gene expression in MSU-stimulated THP-1 macrophages. **g** rhPRG4 (25, 50, 100 and 200μg/ml) treatment reduced TNF-α gene expression in MSU-stimulated THP-1 macrophages. **h** rhPRG4 (50, 100 and 200μg/ml) treatment reduced IL-8 gene expression in MSU-stimulated THP-1 macrophages. **i** rhPRG4 (50, 100 and 200μg/ml) treatment reduced MCP-1 gene expression in MSU-stimulated THP-1 macrophages. **j** rhPRG4 (100 and 200μg/ml) treatment reduced IL-1β production by MSU-stimulated THP-1 macrophages. **k** rhPRG4 (100 and 200μg/ml) treatment reduced TNF-α production by MSU-stimulated THP-1 macrophages. **l** rhPRG4 (200μg/ml) treatment reduced IL-8 production by MSU-stimulated THP-1 macrophages. **m** rhPRG4 (200μg/ml) treatment reduced MCP-1 production by MSU-stimulated THP-1 macrophages

Incubation of THP-1 macrophages with MSU crystals resulted in a significant increase in IL-1β, TNF-α, IL-8 and MCP-1 production by the macrophages at 6 h (*p* < 0.001; *p* = 0.004; *p* < 0.001; *p* < 0.001) (Fig. 2a through d). rhPRG4 and BSM treatments did not significantly alter basal cytokine and chemokine production by THP-1 macrophages (*p > 0.05* for all comparisons). rhPRG4 treatment significantly reduced MSU-induced IL-1β (*p* < 0.001), TNF-α (*p* = 0.003), IL-8 (*p* < 0.001) and MCP-1 (*p* = 0.003) production by THP-1 macrophages at 6 h. In contrast, BSM treatment did not significantly alter MSU-induced cytokines and chemokines production (*p* = 0.305; *p* = 0.365; *p* = 0.964; *p* = 0.998). Media concentrations of IL-1β, IL-8 and MCP-1 were significantly lower in the MSU + rhPRG4 group compared to the MSU + BSM group (*p* < 0.001; *p* = 0.002, *p* = 0.003).

MSU crystals enhanced nuclear NFκB nuclear translocation in THP-1 macrophages compared to untreated control cells (Fig. 2e) (*p* < 0.001). rhPRG4 treatments at 50 and 100 μg/ml significantly reduced MSU stimulated NFκB nuclear translocation in THP-1 macrophages (*p* = 0.039; *p* = 0.008).

MSU crystals induced IL-1β expression compared to untreated macrophages (*p < 0.001*) (Fig. 2f). rhPRG4 (100 and 200μg/ml) treatments reduced IL-1β expression in THP-1 macrophages (*p = 0.002; p = 0.001*). Likewise, MSU crystals significantly induced TNF-α expression compared to untreated macrophages (*p < 0.001*) (Fig. 2g). rhPRG4 (25, 50, 100 and 200μg/ml) treatments reduced TNF-α expression compared to MSU alone group (*p = 0.009*; *p* < 0.001; *p < 0.001; p < 0.001*). MSU crystals significantly induced chemokines IL-8 and MCP-1 expression compared to untreated macrophages (*p < 0.001*) (Fig. 2h and i). rhPRG4 (50, 100 and 200μg/ml) treatments reduced IL-8 expression compared to MSU alone group (*p = 0.031; p = 0.015; p = 0.009*). Similarly, rhPRG4 (50, 100 and 200μg/ml) treatments reduced MCP-1 expression compared to MSU alone group (*p = 0.012*; *p* = 0.002; *p* = 0.001).

At 24 h, treatment with MSU crystals resulted in elevated IL-1β media concentrations compared to controls (*p < 0.001*) (Fig. 2j). rhPRG4 (100 and 200μg/ml) treatment reduced MSU-induced IL-1β production by macrophages (*p* = 0.003; *p* = 0.001). MSU crystals significantly increased TNF-α production by THP-1 macrophages (*p = 0.004*) (Fig. 2k). rhPRG4 (100 and 200μg/ml) treatments significantly reduced MSU-induced TNF-α production by macrophages (*p* = 0.003; *p* = 0.009). MSU crystals significantly induced IL-8 and MCP-1 production by macrophages (*p < 0.001*) (Fig. 2l and m). rhPRG4 (200μg/ml) treatment significantly reduced MSU-stimulated IL-8 and MCP-1 production by macrophages (*p* = 0.004; *p* < 0.001). rhPRG4 (100 and 200μg/ml) alone did not alter the basal levels of cytokines and chemokines (*p* > 0.05 for all comparisons).

**Fig. 3:**
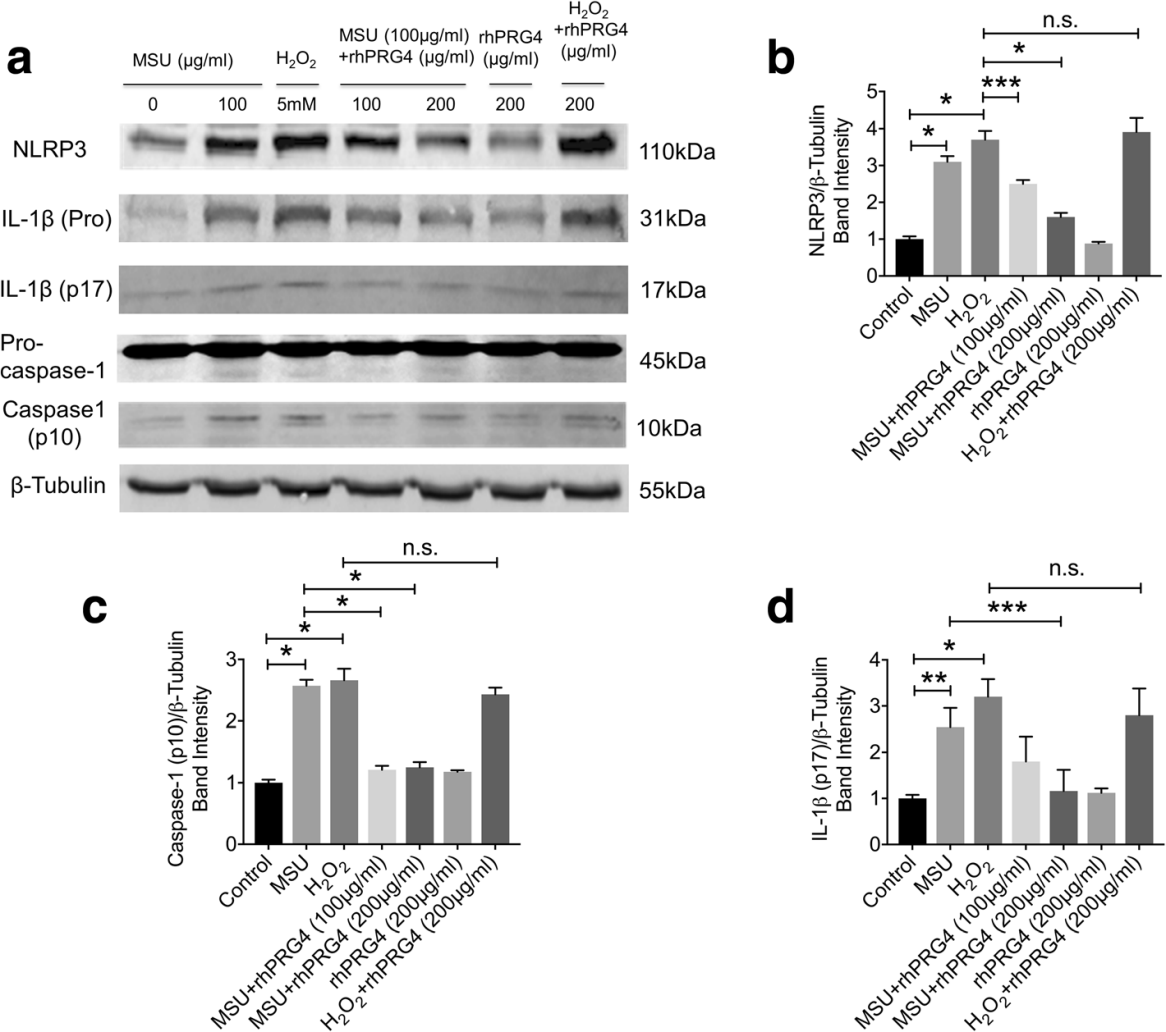
Impact of recombinant human proteoglycan-4 (rhPRG4) treatment on monosodium urate monohydrate (MSU) crystal-induced NLRP3 inflammasome activation in THP-1 macrophages. THP-1 macrophages were treated with 100μg/ml MSU in the absence or presence of rhPRG4 (100 and 200μg/ml) for 12 h. H_2_O_2_ (5 mM) was used as a positive control. Data represent the mean ± S.D. of three independent experiments. *p < 0.001; **p < 0.01; ***p < 0.05; n.s.: non-significant. **a** A representative Western Blot of inflammasome components NLRP3 and procaspase-1, pro-IL-1β, active caspase-1 (p10) and active IL-1β (p17). rhPRG4 treatment reduced NLRP3 induction, procaspase-1 activation and conversion of pro IL-1β to active IL-1β (p17) but did not modify H_2_O_2_ induced inflammasome activation. **b** rhPRG4 (100 and 200μg/ml) treatment reduced NLRP3 protein in MSU-stimulated THP-1 macrophages. **c** rhPRG4 (100 and 200μg/ml) treatment reduced caspase-1 (p10) protein in MSU-stimulated THP-1 macrophages. **d** rhPRG4 (200μg/ml) treatment reduced IL-1β (p17) protein in MSU-stimulated THP-1 macrophages

MSU activated the NLRP3 inflammasome as evidenced by increased cytosolic NLRP3 protein levels in THP-1 macrophages, activated procaspase-1 and increased conversion of proIL-1β to active IL-1β (Fig. 3a). rhPRG4 (100 and 200μg/ml) treatments reduced cytosolic NLRP3 protein levels compared to MSU treatment alone (*p* < 0.05; *p* < 0.001) (Fig. 3b). Similarly, rhPRG4 (100 and 200μg/ml) treatments reduced procaspase-1 activation (*p* < 0.001 for both comparisons; Fig. 3c) and the 200μg/ml treatment level had a lower level of intracellular mature IL-1β (*p* < 0.05; Fig. 3d). rhPRG4 treatment at 200 μg/ml did not alter H_2_O_2_ induced NLRP3 induction, caspase-1 activation or mature IL-1β generation (*p* > 0.05).

### *Prg4*^*−/−*^ peritoneal macrophages demonstrated enhanced MSU crystal intracellular localization at 24 h and IL-1β production compared to *Prg4*^*+/+*^ peritoneal macrophages

**Fig. 4:**
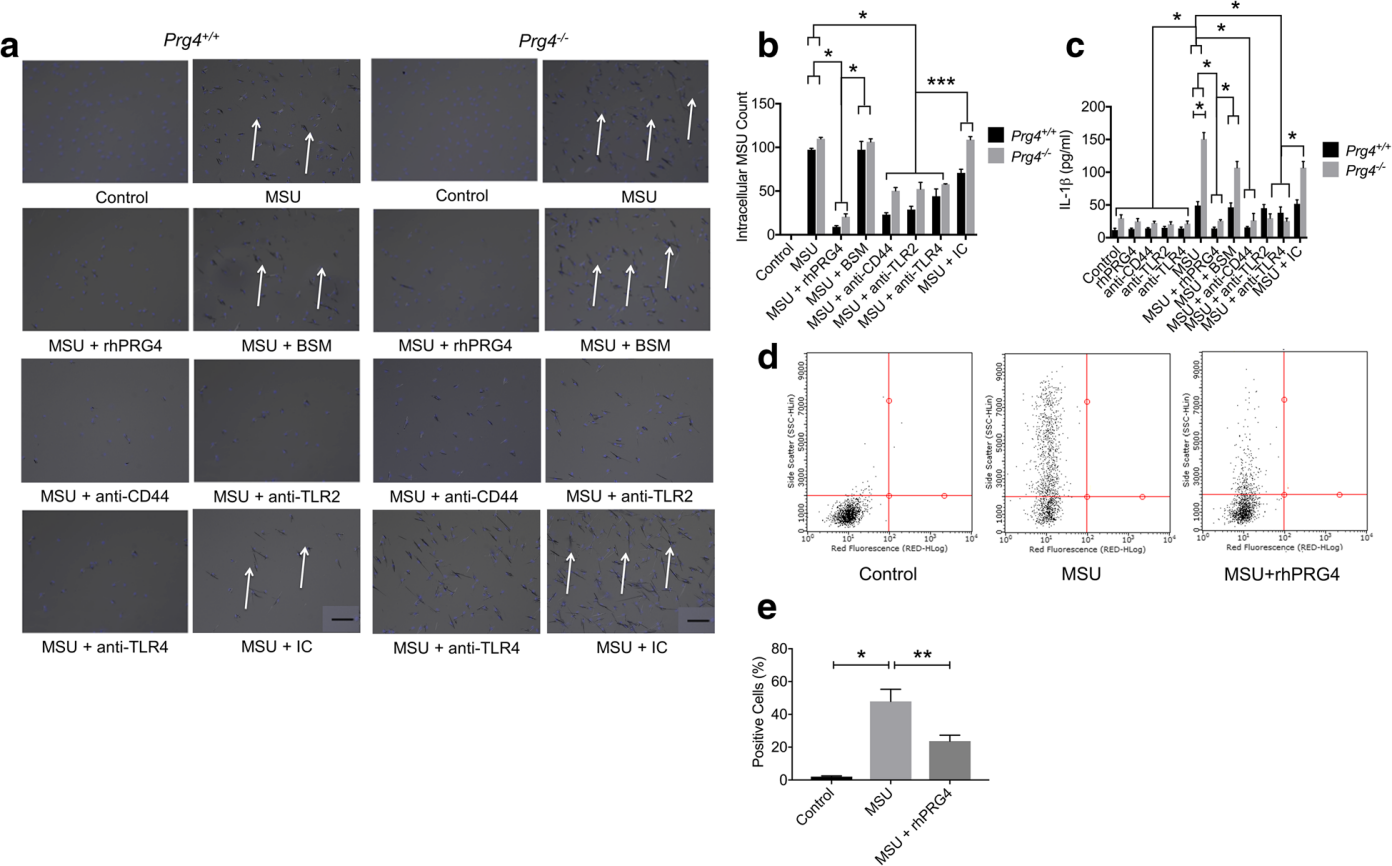
Comparative efficacy of recombinant human proteoglycan-4 (rhPRG4; 100μg/ml), bovine submaxillary mucin (BSM; 25μg/ml), anti-CD44, anti-toll-like receptor 2 (TLR2), anti-toll-like receptor 4 (TLR4) and isotype control (IC) antibodies (2μg/ml for all antibodies) treatments on phagocytosis of monosodium urate monohydrate (MSU; 100μg/ml) crystals by primary peritoneal murine macrophages from *Prg4*^*+/+*^ and *Prg4*^*−/−*^ mice following a 4-h incubation and production of interleukin-1 beta (IL-1β). Data represent the mean ± S.D. of four independent experiments. *p < 0.001; **p < 0.01; ***p < 0.05. Scale = 50 μm. **a** Representative images of DAPI-stained peritoneal macrophages from *Prg4*^*+/+*^ and *Prg4*^*−/−*^ mice with all treatments. Arrows point to MSU crystals localized intracellularly. rhPRG4, anti-CD44, ani-TLR2 and anti-TLR4 treatments reduced MSU phagocytosis by *Prg4*^*+/+*^ and *Prg4*^*−/−*^ peritoneal macrophages. **b** Intracellular count of MSU crystals in *Prg4*^*+/+*^ and *Prg4*^*−/−*^ peritoneal macrophages. A specific effect for rhPRG4, anti-CD44, anti-TLR2 and anti-TLR4 treatments was observed. **c** rhPRG4 and anti-CD44 antibody treatments reduced IL-1β production by *Prg4*^*+/+*^ and *Prg4*^*−/−*^ peritoneal macrophages. rhPRG4, anti-CD44, anti-TLR2 and anti-TLR4 treatments reduced IL-1β production by *Prg4*^*−/−*^ peritoneal macrophages. **d** Representative flow cytometry of MSU-treated macrophages in the absence or presence of rhPRG4 (100μg/ml) for 6 h. **e** rhPRG4 treatment reduced MSU phagocytosis at 6 h

**Fig. 5:**
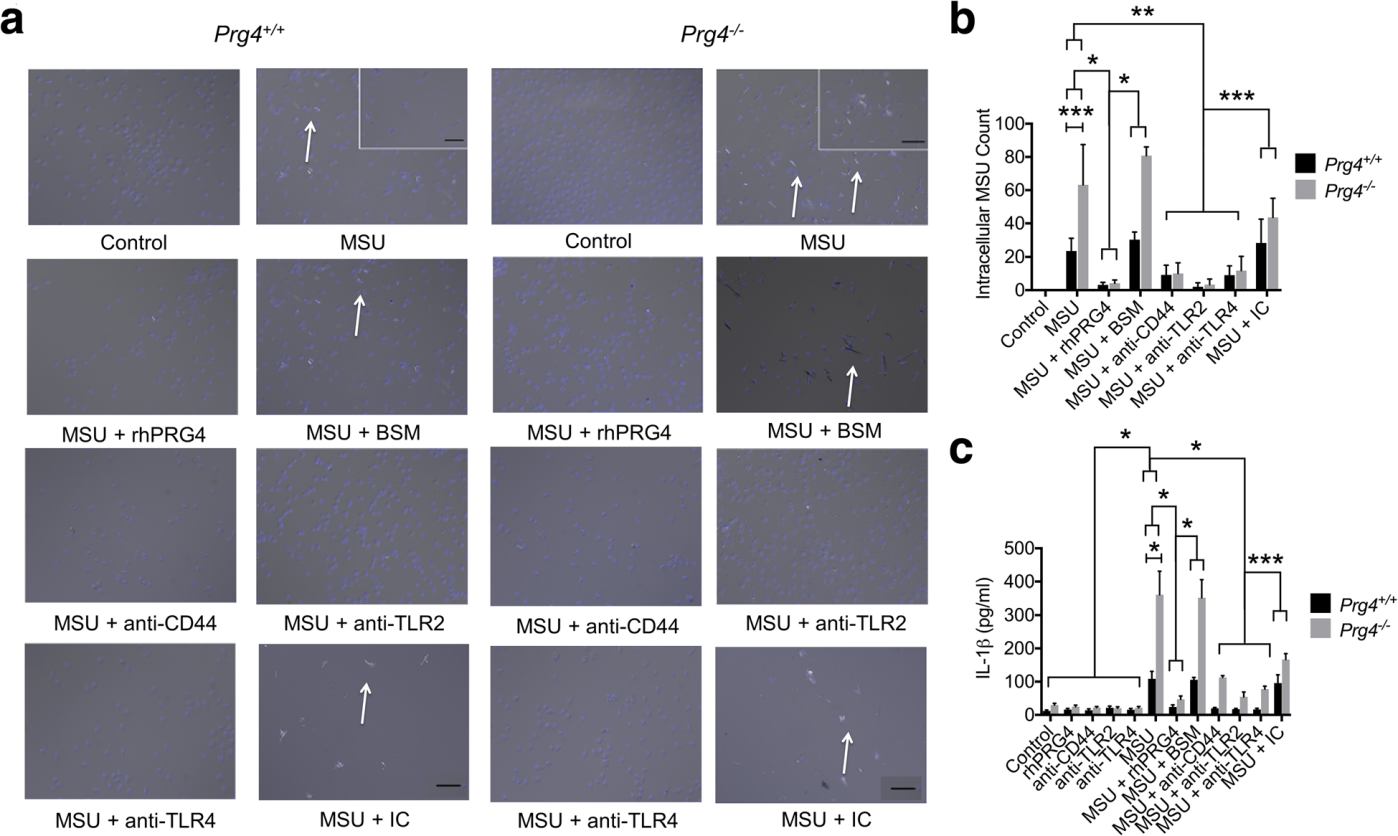
Comparative efficacy of recombinant human proteoglycan-4 (rhPRG4; 100μg/ml), bovine submaxillary mucin (BSM; 25μg/ml), anti-CD44, anti-toll-like receptor 2 (TLR2), anti-toll-like receptor 4 (TLR4) and isotype control (IC) antibodies (2μg/ml for all antibodies) treatments on phagocytosis of monosodium urate monohydrate (MSU; 100μg/ml) crystals by primary peritoneal murine macrophages from *Prg4*^*+/+*^ and *Prg4*^*−/−*^ mice following a 24-h incubation and production of interleukin-1 beta (IL-1β). Data represent the mean ± S.D. of five independent experiments. *p < 0.001; **p < 0.01; ***p < 0.05. Scale = 50 μm. **a** Representative images of DAPI-stained peritoneal macrophages from *Prg4*^*+/+*^ and *Prg4*^*−/−*^ mice with all treatments. Arrows point to MSU crystals localized intracellularly. rhPRG4, anti-CD44, ani-TLR2 and anti-TLR4 treatments reduced MSU phagocytosis by *Prg4*^*+/+*^ and *Prg4*^*−/−*^ peritoneal macrophages. **b** Intracellular count of MSU crystals in *Prg4*^*+/+*^ and *Prg4*^*−/−*^ peritoneal macrophages. A specific effect for rhPRG4, anti-CD44, anti-TLR2 and anti-TLR4 treatments was observed. **c** rhPRG4, anti-CD44, anti-TLR2 and anti-TLR4 treatments reduced IL-1β production by *Prg4*^*+/+*^ and *Prg4*^*−/−*^ peritoneal macrophages

Phagocytosis of MSU crystals by *Prg4*^*−/−*^ and *Prg4*^*+/+*^ peritoneal macrophages are shown in Figs. 3 and 4. MSU crystals appeared to have been internalized by macrophages from both genotypes as early as 4 h and continued to be detected up to 24 h. At 4 h, there was no significant difference in intracellular MSU crystal count between *Prg4*^*−/−*^ and *prg4*^*+/+*^ macrophages (*p = 0.739*) (Fig. 4b). In contrast, we have observed a significantly higher number of MSU crystals in *Prg4*^*−/−*^ macrophages compared to *Prg4*^*+/+*^ macrophages (*p = 0.019*) at 24 h (Fig. 5b). *Prg4*^*−/−*^ peritoneal macrophages secreted significantly higher quantities of IL-1β compared to *Prg4*^*+/+*^ peritoneal macrophages at 4 h (*p* < 0.001) (Fig. 4c) and 24 h (*p* < 0.001) (Fig. 5c).

### Neutralization of CD44, TLR2 and TLR4 receptors reduced MSU crystal phagocytosis and downstream IL-1β production in primary murine peritoneal macrophages similar to rhPRG4

Representative images of DAPI-stained *Prg4*^*−/−*^ and *Prg4*^*+/+*^ peritoneal macrophages showing the impact of rhPRG4, BSM, anti-CD44, anti-TLR2, anti-TLR4 and IC antibodies treatments at 4 and 24 h are shown in Figs. 4a and 5a. rhPRG4 significantly reduced MSU crystal uptake by peritoneal macrophages from both genotypes at 4 and 24 h (*p < 0.001* for all comparisons) (Fig. 4b and 5b). In contrast, BSM treatment had no significant effect on MSU phagocytosis (*p > 0.05* for all comparisons). Neutralization of CD44, TLR2 and TLR4 receptors significantly reduced MSU phagocytosis in *Prg4*^*−/−*^ and *Prg4*^*+/+*^ peritoneal macrophages at 4 and 24 h incubations (*p < 0.01* for all comparisons). Treatment with IC did not alter MSU phagocytosis by *Prg4*^*−/−*^ and *Prg4*^*+/+*^ macrophages at 4 h and crystal phagocytosis by *Prg4*^*+/+*^ macrophages at 24 h (*p > 0.05* for all comparisons). We observed a non-specific effect of antibody treatment on MSU uptake by *Prg4*^*−/−*^ peritoneal macrophages at 24 h. MSU phagocytosis in the MSU + IC group was significantly lower than MSU phagocytosis in MSU alone group (*p < 0.05*). The percent positive cells with increased side scattering due to MSU phagocytosis was significantly reduced with rhPRG4 treatment at 6 h (*p* < 0.001; Fig. 4e).

Media concentrations of IL-1β were significantly lower with rhPRG4, anti-CD44, anti-TLR2 and anti-TLR4 treatments at 4 and 24 h utilizing *Prg4*^*−/−*^ peritoneal macrophages (*p < 0.0.5* for all comparisons) (Fig. 4c and 5c). Likewise, media concentrations of IL-β were significantly lower with rhPRG4, anti-CD44, anti-TLR2 and anti-TLR4 treatments at 24 h utilizing *Prg4*^*+/+*^ macrophages. Only rhPRG4 and anti-CD44 treatments significantly reduced IL-1β production in *Prg4*^*+/+*^ macrophages at 4 h (*p < 0.01*). Neither anti-TLR2 nor anti-TLR4 significantly reduced IL-1β production in *Prg4*^*+/+*^ macrophages at 4 h (*p > 0.05*). We did not detect a non-specific effect by BSM or IC treatments utilizing *Prg4*^*+/+*^ peritoneal macrophages at 4 and 24 h or at 4 h utilizing *Prg4*^*−/−*^ macrophages. We observed a non-specific effect of antibody treatment on MSU-stimulated IL-1β production in *Prg4*^*−/−*^ peritoneal macrophages at 24 h. IL-1β concentrations in the MSU + IC group were significantly lower than corresponding concentrations in the MSU alone group (*p < 0.05*). Antibody-mediated neutralization of the CD44, TLR2 and TLR4 receptors yielded similar efficacy in reducing MSU crystal phagocytosis and IL-1β production similar to rhPRG4 treatment.

### rhPRG4 preferentially colocalized with CD44 receptor on *Prg4*^*−/−*^ peritoneal macrophages

**Fig. 6:**
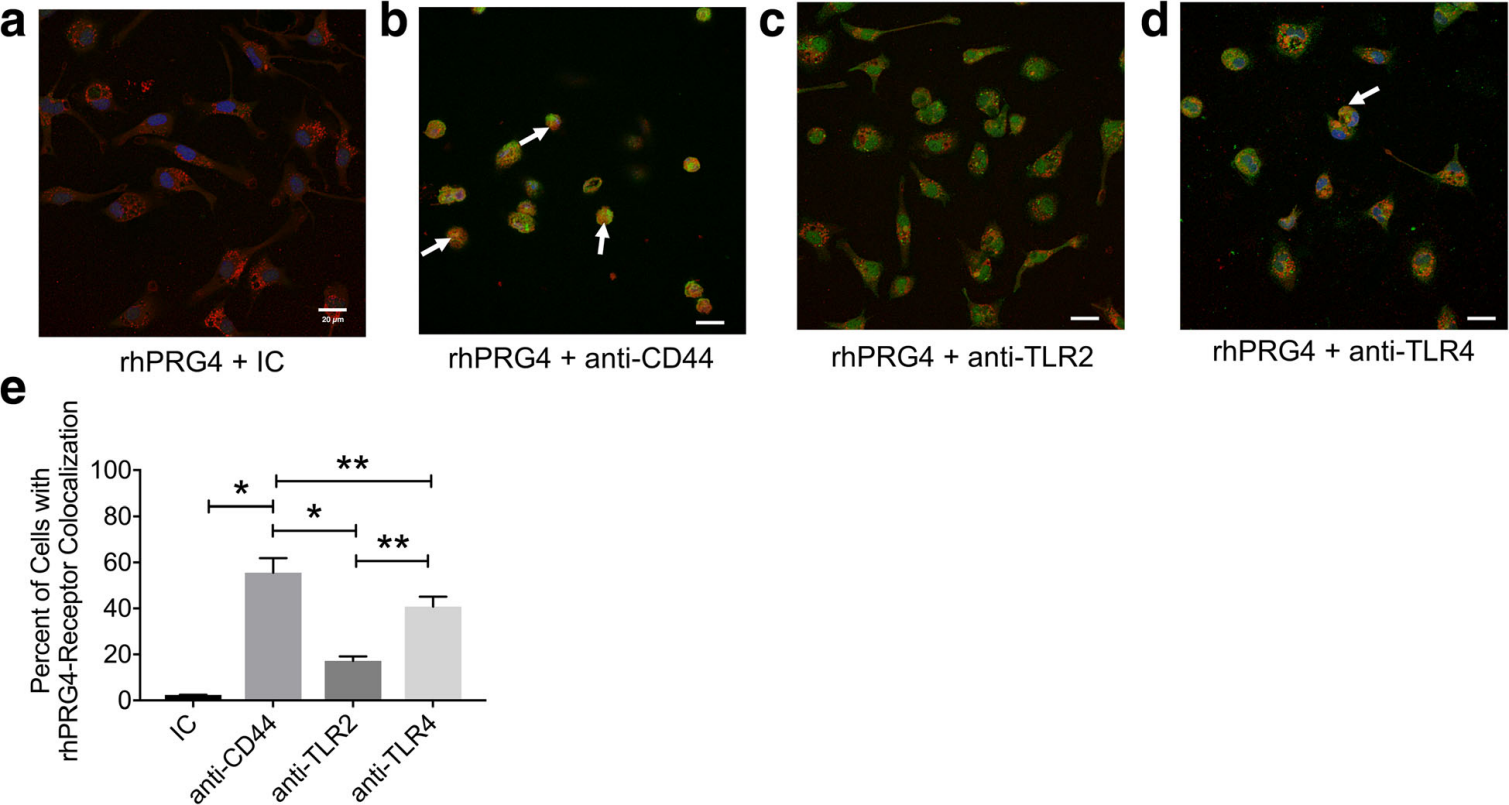
Colocalization of rhodamine-labeled recombinant human proteoglycan-4 (rhPRG4) (red) and isotype control (IC), CD44 (probed using anti-CD44), toll-like receptor 2 (TLR2) (probed using anti-TLR2) or toll-like receptor 4 (TLR4) (probed using anti-TLR4) in peritoneal *Prg4*^*−/−*^ murine macrophages. Cells were incubated with rhodamine-rhPRG4 for 2 h followed by cell fixation and permeabilization. Following receptor probing, cells were incubated with Alexa Fluor 488 conjugated secondary antibody (green) and counterstained with DAPI (blue). Arrows point to co-localization of rhPRG4 with respective receptors. Quantitative colocalization analysis was performed using Pearson’s Correlation Coefficient and a cutoff of r^2^ > 0.5 was used to indicate positive colocalization. The percentage of cells with positive colocalization was determined and at least 100 cells were examined for each treatment condition. Data represent the mean ± S.D. of three independent experiments. Median colocalization images are presented. *p < 0.001; **p < 0.01; ***p < 0.05. Scale = 20μm. **a** Representative image of rhodamine-rhPRG4 treated *Prg4*^*−/−*^ macrophages and probed with IC antibody. **b** Representative image of rhodamine-rhPRG4 treated *Prg4*^*−/−*^ macrophages and probed with anti-CD44 antibody. **c** Representative image of rhodamine-rhPRG4 treated *Prg4*^*−/−*^ macrophages and probed with anti-TLR2 antibody. **d** Representative image of rhodamine-rhPRG4 treated *Prg4*^*−/−*^ macrophages and probed with anti-TLR4 antibody. **e** Colocalization of rhPRG4 and CD44 was higher compared to rhPRG4 and TLR2 colocalization and rhPRG4 and TLR4 colocalization

Representative colocalization images of rhPRG4 and CD44, TLR2 and TLR4 receptors are shown in Fig. 6b, c and d, respectively. We have qualitatively observed more colocalization of rhPRG4 with CD44 compared to TLR2 or TLR4 receptors. Furthermore, internalization of rhPRG4 with CD44 was also observed. The mean percentage of peritoneal macrophages that were positive for rhPRG4 and CD44 colocalization was 55.56% compared to 17.21% for rhPRG4 and TLR2 colocalization and 40.78% for rhPRG4 and TLR4 colocalization. rhPRG4 and CD44 colocalization was significantly higher than rhPRG4 and TLR2 colocalization (*p < 0.001*) and rhPRG4 and TLR4 colocalization (*p < 0.01*) (Fig. 6e). Additionally, rhPRG4 and TLR4 colocalization was significantly higher than rhPRG4 and TLR2 colocalization (*p < 0.01*).

### rhPRG4 treatment normalized weight bearing and reduced SF myeloperoxidase activity

**Fig. 7:**
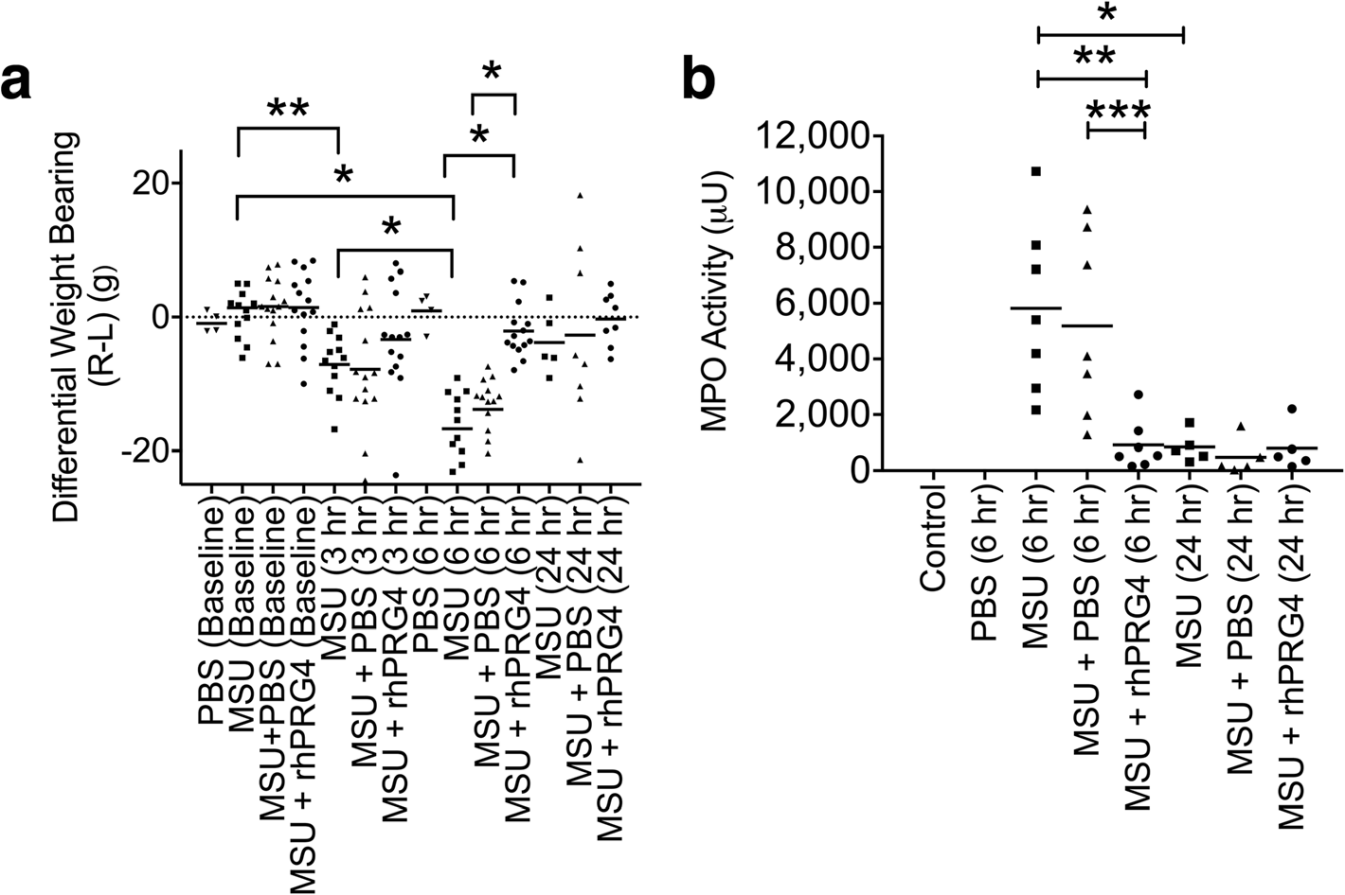
Impact of recombinant human proteoglycan-4 (rhPRG4) treatment on differential weight bearing (Right hind limb – Left hind limb; R-L) and synovial fluid (SF) lavage myeloperoxidase (MPO) activity following intra-articular administration of monosodium urate monohydrate (MSU) crystals (50μL; 2.5 mg/mL) in the right knee joint of male Lewis rats followed by intra-articular treatments with rhPRG4 (1 mg/mL; 50 μL) or PBS (50 μL) at 1 h following MSU administration, or no treatment. Differential weight bearing was measured at 3 h (*n* = 12 in MSU alone and *n* = 14 in rhPRG4 or PBS treatments), 6 h (*n* = 12 in MSU alone and *n* = 14 in rhPRG4 or PBS treatments) and 24 h (*n* = 5 in MSU alone and *n* = 7 in rhPRG4 or PBS treatments) following MSU administration. SF lavage MPO activities were determined at 6 h (*n* = 7 in each group) and 24 h (*n* = 5 in each group) following MSU administration. Data are presented as a scatterplot with the mean value highlighted. *p < 0.001; **p < 0.01; ***p < 0.05. **a** rhPRG4 treatment decreased MSU-induced differential weight bearing at 6 h compared to PBS treatment or no treatment. **b** rhPRG4 treatment reduced MSU-induced elevation in SF lavage MPO activity at 6 h compared to PBS treatment or no treatment

Differential weight bearing in the MSU alone group at 3 and 6 h was significantly lower than the differential weight bearing at baseline (*p = 0.007*; *p < 0.001*) (Fig. 7a). Additionally, the differential weight bearing in the MSU alone group at 6 h was significantly lower than corresponding values at 3 h (*p < 0.001*). At 3 h, there was no significant change in differential weight bearing in PBS or rhPRG4-treated animals vs. MSU alone (*p = 0.968*; *p* = 0.421). At 6 h, rhPRG4 treatment normalized weight bearing as differential weight bearing values in rhPRG4-treated animals were significantly higher than corresponding values in MSU alone or MSU + PBS groups (*p < 0.001* for both comparisons). There was no observed effect for PBS treatment compared to MSU alone (*p = 0.2770*). At 24 h, weight bearing in all experimental groups returned to baseline. There was no detectable SF lavage MPO activity in untreated control and PBS-injected animals (Fig. 7b). At 6 h, mean SF lavage MPO activity in rhPRG4 treated animals was significantly lower than corresponding value in PBS-treated and MSU alone animals (*p* = 0.018; *p* = 0.007). There was no significant difference in mean SF lavage MPO activity between MSU alone and MSU + PBS groups at 6 h (*p = 0.894*). At 24 h, there were no significant differences among the different experimental groups (*p* > 0.05 for all comparisons).

## Discussion

In this work, we studied the activation of macrophages from human and murine origins by MSU crystals and evaluated the consequence of rhPRG4 treatment on MSU induced inflammation. MSU crystals were phagocytosed by macrophages in a time-dependent manner resulting in an increase in NFκB p65 subunit nuclear translocation, induction of NLRP3 protein, activation of procaspase-1 enzyme and conversion of proIL-1β to mature IL-1β. The downstream effects included the induction of the expression and production of IL-1β, TNF-α, IL-8 and MCP-1 over a 24-h period. Concentrations of these cytokines and chemokines produced by macrophages subsequent to MSU crystal stimulation were detectable as early as 4 h in murine macrophages and 6 h in human macrophages and remained elevated over a 24-h period. The induction of cytokines and chemokines gene expression and production secondary to MSU stimulation was most pronounced for IL-1β and IL-8. This observation is in agreement with previous reports demonstrating enhanced IL-1β and IL-8 expression and production by macrophages in vitro [22, 38]. PRG4’s protein core is 1404 amino acid long with *N* and *C* termini and a central mucin domain that is heavily glycosylated via O-linked β(1–3) Gal-GalNAc oligosaccharides, and is configured to form a nanofilm that exerts repulsive forces, and provides the basis for its anti-adhesive and lubricating properties [39]. We studied the efficacy of rhPRG4 against submaxillary mucin to evaluate the extent of any non-specific biophysical effect that may have resulted from the mucinous nature of rhPRG4 [40]. Contrary to rhPRG4, submaxillary mucin showed no appreciable effect on MSU phagocytosis or MSU-induced inflammation in both murine and human macrophages. rhPRG4 demonstrated a time-dependent and concentration-dependent inhibition of MSU crystal phagocytosis and NFκB nuclear activation as well as a reduction in NLRP3 inflammasome activation. rhPRG4 dose-dependently reduced MSU-induced gene expression and production of IL-1β, TNF-α, IL-8 and MCP-1. TNF-α and IL-8 gene expression and production were most susceptible to the inhibitory effect of rhPRG4. Overall, rhPRG4 exhibited an anti-inflammatory activity at physiologically relevant concentrations that have been previously reported in SF aspirates from normal subjects and from patients with OA [41]. The inhibitory effect of rhPRG4 on inflammasome activation was specific for uric acid crystals as rhPRG4 failed to inhibit inflammasome activation due to the generation of reactive oxygen species.

PRG4 plays a homeostatic role in the articular joint with an established role in regulating synovial overgrowth and preserving cartilage integrity [42, 43]. Findings in joints from *Prg4*^*−/−*^ mice include synovial hyperplasia, cartilage surface fibrillations and chondrocyte apoptosis [34, 43–45]. These pathological changes are mostly irreversible even with restoration of PRG4 expression [45]. Interestingly, synoviocytes isolated from knee synovial tissues of *Prg4*^*−/−*^ mice exhibit a proinflammatory phenotype characterized by upregulation of CD44 receptor and enhanced basal and cytokine induced proliferation compared to synoviocytes isolated from wild type animals [26]. We have isolated peritoneal macrophages from *Prg4*^−/−^ and *Prg4*^*+/+*^ animals and studied their time-dependent MSU crystal phagocytosis and resultant IL-1β secretion. While we did not observe a marked difference in the extent of MSU internalization by macrophages from *Prg4* null and competent animals at 4 h, MSU crystals have accumulated in the *Prg4*^*−/−*^ macrophages compared to the wildtype counterparts by 24 h, with approximately 3 times the number of intracellular MSU crystals in *Prg4*^−/−^ macrophages compared to wildtype macrophages. The accumulation of urate crystals inside *Prg4*^−/−^ macrophages may be due to enhanced phagocytosis over time or impaired degradation of intracellular urate crystals in the or a combination of both. The uptake of MSU by *Prg4*^−/−^ and wild type macrophages was reduced by rhPRG4 treatment at 4 h and this effect was sustained over 24 h. We have also observed an enhanced MSU stimulated IL-1β production by *Prg4*^−/−^ macrophages compared to wild type macrophages with approximately 3-fold increase in IL-1β production by knockout macrophages in relation to wild type macrophages after incubation for 4 h and remained up to 24 h. This suggests that *Prg4* null macrophages are primed to the inflammation triggering effect of MSU crystals, which can be rationalized by the low grade inflammatory phenotype of *Prg4*^−/−^ mice [46]. Our combined findings support that PRG4 may have an anti-inflammatory biological role in regulating the activation of tissue macrophages by danger signals e.g. MSU crystals.

To gain more insight into the molecular target of rhPRG4 that mediates its anti-phagocytic and anti-inflammatory effects, we have conducted comparative efficacy studies of rhPRG4 against antibody-mediated neutralization of CD44, TLR2 and TLR4 receptors using MSU challenged *Prg4*^*−/−*^ and wildtype macrophages. We have also performed colocalization studies to identify the putative receptor target on the surface of macrophages. Phagocytosis of MSU crystals by human and murine macrophages and downstream IL-1β production were reversed by CD44, TLR2 and TLR4 receptor neutralization. The neutralization of these receptors resulted in a similar anti-inflammatory efficacy to that of rhPRG4. The inhibitory effect of TLR2 and TLR4 neutralizing antibodies supports a role for TLR2 and TLR4 in mediating the initial steps of gout pathogenesis [5]. CD44 is a transmembrane receptor with an important role in inflammation [47]. In addition to regulating cellular migration and adhesion, CD44 receptor has a role in regulating cell signaling pathways owing to its ability to regulate signaling protein assembly [47]. Macrophage CD44 receptor was shown to mediate complement-dependent and independent phagocytosis [48, 49]. In addition to its direct phagocytic role, CD44 was shown to negatively regulate TLR stimulation [32, 50, 51]. Neutralization of CD44 using a CD44-specific antibody was shown to reduce NFκB nuclear translocation and proinflammatory cytokine expression and production by macrophages in response to TLR2 ligand stimulation [32]. The anti-phagocytic activity of CD44 receptor neutralization, shown in our murine macrophage experiments, provides evidence that CD44 may act as a regulator of MSU induced inflammation in macrophages. The involvement of CD44 is further highlighted by preferential colocalization of rhPRG4 with CD44 compared to TLR2 or TLR4 on the surface of macrophages, likely indicating that the effect of rhPRG4 is based on its CD44 interaction. The involvement of CD44 in mediating rhPRG4’s effect is further supported by the higher binding affinity that rhPRG4 exhibit against CD44 compared to either TLR2 or TLR4 [26, 28, 29]. The involvement of CD44 receptor in the function of rhPRG4 cannot definitively rule out other accessory mechanisms. The complexity of the interaction between PRG4 and cell surfaces is highlighted by the unique and multifunctional structure of PRG4. PRG4 was shown to bind to L-selectin in a glycosylation-dependent manner [52, 53]. Additionally, PRG4 amino terminal domains are homologous to somatomedin B domain of vitronectin and the carboxy terminal contains a hemopexin domain and may mediate surface binding of the protein [54].

IL-1β plays a pivotal role in mediating gouty inflammation and IL-1 inhibitors were shown to relieve pain and inflammation in rodent models and in clinical experiences [55–57]. IL-1 inhibitors do not interfere with MSU phagocytosis by macrophages and other cells in the joint and thus do not block the resultant expression and production of proinflammatory cytokines and chemokines. IL-1 inhibitors block the autocrine and paracrine effects of locally produced IL-1β and hence the downstream inflammatory cascade. rhPRG4 works at an earlier point in the gout inflammatory pathway by reducing MSU phagocytosis. The mechanism of action of rhPRG4 results in an indirect IL-1 antagonist effect, via reducing IL-1β production and hence attenuating its role in driving gout pathogenesis. We thus also studied crystal-induced inflammation in vivo in the rat. Intra-articular administration of MSU resulted in a spike in MPO activity at 6 h that gradually resolved by 24 h. MPO is abundantly expressed and released from neutrophils and is a marker of neutrophil tissue infiltration and oxidative stress [58, 59]. Specific to gout, MPO activity in articular joint tissues increased and has been previously correlated with neutrophil influx in a mouse model of crystal-induced inflammation [21]. rhPRG4 treatment reduced joint inflammation following MSU challenge. Hypernociception was evident following MSU challenge and the time course of mechanical allodynia mirrored joint inflammation. This is in accordance with previous reports that demonstrated that synovial tissue COX2 gene expression and associated mechanical allodynia were significantly increased following MSU administration in rat knee or ankle joints [60–62]. This novel in vivo anti-nociceptive and anti-inflammatory efficacy of rhPRG4 builds upon previously reported efficacy of rhPRG4 in pre-clinical PTOA models [63–66] and provides a rationale for further investigation of rhPRG4’s efficacy as a treatment for acute gout.

Our study was limited by the brief duration of inflammation that was observed in the rat model, which might have limited our ability to comprehensively characterize the efficacy of rhPRG4 in vivo. Future study will use a higher dose of MSU crystals, which has been recently optimized [67]. Additionally, we have not studied the inflammatory effect of MSU challenge in *Prg4*^*+/+*^ and *Prg4*^*−/−*^ mice in vivo*.* In our experiments, we observed a reduction in IL-1β gene expression and secreted IL-1β levels at the 100 μg/ml level following a 6-h incubation period. Alternatively, we detected an inhibitory effect of rhPRG4 on intracellular mature IL-1β at 200 μg/ml following a 12-h incubation period*.* Collectively, these observations demonstrate the anti-inflammatory potency of rhPRG4.

## Conclusion

rhPRG4 inhibited MSU crystal phagocytosis by human and murine macrophages, reduced NFκB p65 subunit nuclear translocation and downstream proinflammatory cytokines and chemokines expression and production in vitro. Neutralization of CD44, TLR2 and TLR4 receptors on murine macrophages yielded similar efficacy to rhPRG4, namely a reduction in MSU phagocytosis and downstream IL-1β production. rhPRG4 demonstrated a higher binding affinity and colocalization with the CD44 receptor compared to TLR2 or TLR4 receptors. These findings suggest that the CD44 receptor may play a role in regulating MSU phagocytosis by macrophages and that rhPRG4’s efficacy is partly due to its CD44-based mechanism. Intra-articular administration of rhPRG4 reduced MPO activity and normalized weight bearing in a rat model.

## Abbreviations

ANOVA: Analysis of Variance
CD44 receptor: Cluster determinant 44 receptor
Ct: Cycle Threshold
ELISA: Enzyme-linked immunosorbent assay
FBS: Fetal bovine serum
GAPDH: Glyceraldehyde-3-phosphate dehydrogenase
HEPES: (4-(2-hydroxyethyl)-1-piperazineethanesulfonic acid)
IκBα: Inhibitor kappa B alpha
IL-1β: Interleukin-1 beta
IL-8: Interleukin-8
MCP-1: Monocyte chemoattractant protein-1
MSU: Monosodium urate monohydrate
NFκB: Nuclear Factor Kappa B
OA: Osteoarthritis
PBS: Phosphate-buffered saline
PRG4: Proteoglycan-4
qPCR: Quantitative PCR
RA: Rheumatoid arthritis
rhPRG4: Recombinant human PRG4
RPMI medium: Roswell Park Memorial Institute medium
S.D.: Standard deviation
TBS-T: Tris-buffered saline Tween 20
TLR2: Toll-like receptor 2
TLR4: Toll-like receptor 4
TNFα: Tumor Necrosis Factor alpha

## References

[1] Pascual E, Addadi L, Andres M, Sivera F (2015). Mechanisms of crystal formation in gout-a structural approach. Nat Rev Rheumatol.

[2] Bitik B, Akif ÖM (2014). An old disease with new insights: update on diagnosis and treatment of gout. Eur J Rheumatol.

[3] Stewart S, Dalbeth N, Vandel A, Rome K (2016). The first metatarsophalangeal joint in gout: a systematic review and meta-analysis. BMC Musculoskeletal Disord.

[4] Busso N, So A (2010). Mechanisms of inflammation in gout. Arthritis Res Ther.

[5] Liu-Bryan R, Scott P, Sydalske A, Rose DM, Terkeltaub R (2005). Innate immunity conferred by toll-like receptors 2 and 4 and myeloid differentiation factor 88 expression is pivotal to monosodium urate monohydrate crystal-induced inflammation. Arthritis Rheum.

[6] Scott P, Mia H, Viriyakosol S, Terkeltaub R, Liu-Bryan R (2006). Engagement of CD14 mediates the inflammatory potential of monosodium urate crystals. J Immunol.

[7] Martin WJ, Walton M, Harper J (2009). Resident macrophages initiating and driving inflammation in a monosodium urate monohydrate crystal-induced murine peritoneal model of acute gout. Arthritis Rheum.

[8] So AK, Martinon F (2017). Inflammation in gout: mechanisms and therapeutic targets. Nat Rev Rheumatol.

[9] Holzinger D, Nippe N, Vogi T, Marketon K, Mysore V (2014). Myeloid-related proteins 8 and 14 contribute to monosodium urate monohydrate crystal-induced inflammation in gout. Arthritis Rheumatol.

[10] Joosten LA, Netea MG, Mylona E, Koenders MI, Malireddi RK (2010). Engagement of fatty acids with toll-like receptor 2 drives interleukin-1β production via the ACS/caspases 1 pathway in monosodium urate monohydrate crystal-induced gouty arthritis. Arthritis Rheum.

[11] Martinon F, Mayor A, Tschopp J (2009). The inflammasomes: guardians of the body. Annu Rev Immunol.

[12] Choi AJ, Ryter SW (2014). Inflammasomes: molecular regulation and implications for metabolic and cognitive diseases. Mol Cells.

[13] He Y, Hara H, Nunez G (2016). Mechanism and regulation of NLRP3 inflammasome activation. Trends Biochem Sci.

[14] Chen CJ, Shi Y, Hearn A, Firzgerald K, Golenbock D (2006). MyD88-dependent IL-1 receptor signaling is essential for gouty inflammation stimulated by monosodium urate crystals. J Clin Invest.

[15] Nishimura A, Akahoshi T, Takahashi M, Takagishi K, Itoman M (1997). Attenuation of monosodium urate crystal-induced arthritis in rabbits by a neutralizing antibody against interleukin-8. J Leukoc Biol.

[16] Pope RM, Tschopp J (2007). The role of interleukin-1 and the inflammasome in Gout. Arthritis Rheum.

[17] Pessler F, Mayer CT, Jung SM, Behrens EM, Dai L (2008). Identification of novel monosodium urate crystal regulated mRNAs by transcript profiling of dissected murine air pouch membranes. Arthritis Res Ther.

[18] Castelblanco M, Lugrin J, Ehirchiou D, Nasi S, Ishii I, et al. Hydrogen sulfide inhibits the NLRP3 inflammasome and reduces cytokine production both in vitro and in a mouse model of inflammation. J Biol Chem. 2017; 10.1074/jbc.M117.806869. 10.1074/jbc.M117.806869 PMC5818172 29279328

[19] Ghaemi-Oskouie F, Shi Y (2011). The role of uric acid as an endogenous danger signal in immunity and inflammation. Curr Rheumatol Rep.

[20] Martinon F, Petrilli V, Mayor A, Tardivel A, Tschopp J (2006). Gout-associated uric acid crystals activate the NALP3 inflammasome. Nature.

[21] Amaral F, Costa VV, Tavares LD, Sachs D, Coelho FM (2012). NLRP3 inflammasome-mediated neutrophil recruitment and Hypernociception depend on leukotriene B(4) in a murine model of gout. Arthritis Rheum.

[22] Pazar B, Ea HK, Narayan S, Kolly L, Bagnoud N (2011). Basic calcium phosphate crystals induce monocyte/macrophage IL-1β secretion through the NLRP3 inflammasome in vitro. J Immunol.

[23] Jay GD, Britt DE, Cha CJ (2000). Lubricin is a product of megakaryocyte stimulating factor gene expression by human synovial fibroblasts. J Rheumatol.

[24] Jay GD, Tantravahi U, Britt DE, Barrach HJ, Cha CJ (2001). Homology of lubricin and superficial zone protein (SZP): products of megakaryocyte stimulating factor (MSF) gene expression by human synovial fibroblasts and articular chondrocytes localized to chromosome 1q25. J Orthop Res.

[25] Flannery CR, Hughes CE, Schumacher BL, Tudor D, Aydelotte MB (1999). Articular cartilage superficial zone protein (SZP) is homologous to megakaryocyte stimulating factor precursor and is a multifunctional proteoglycan with potential growth-promoting cytoprotective, and lubricating properties in cartilage metabolism. Biochem Biophys Res Commun.

[26] Al-Sharif A, Jamal M, Zhang L, Larson K, Schmidt TA (2015). Lubricin/proteoglycan 4 binding to CD44 receptor: a mechanism of lubricin’s suppression of proinflammatory cytokine induced synoviocyte proliferation. Arthritis Rheumatol.

[27] Alquraini A, Jamal M, Zhang L, Schmidt TA, Jay GD (2017). The autocrine role of proteoglycan-4 (PRG4) in modulating osteoarthritic synoviocyte proliferation and expression of matrix degrading enzymes. Arthritis Res Ther.

[28] Iqbal SM, Leonard C, Regmi SC, De Rantere D, Tailor P (2016). Lubricin/proteoglycan 4 binds to and regulates the activity of toll-like receptors in vitro. Sci Rep.

[29] Alquraini A, Garguilo S, D’Souza G, Zhang LX, Schmidt TA (2015). The interaction of lubricin/proteoglycan-4 (PRG4) with toll-like receptors 2 and 4: an anti-inflammatory role of PRG4 in synovial fluid. Arthritis Res Ther.

[30] Park EK, Jung HS, Yang HI, Yoo MC, Kim C (2007). Optimized THP-1 differentiation is required for the detection of response to weak stimuli. Inflamm Res.

[31] Samson ML, Morrison S, Masala N, Sullivan BD, Sullivan DA (2014). Characterization of full-length recombinant human proteoglycan 4 as an ocular surface boundary lubricant. Exp Eye Res.

[32] Qadri M, Almadani S, Jay GD, Elsaid KA (2018). Role of CD44 in regulating toll-like receptor 2 (TLR2) activation of human macrophages and downstream expression of proinflammatory cytokines. J Immunol.

[33] Livak KJ, Schmittgen TD (2001). Analysis of relative gene expression data using real-time quantitative PCR and the 2(−Delta Delta C(T)) method. Methods.

[34] Rhee DK, Marcelino J, Baker M, Gong Y, Smits P (2005). The secreted glycoprotein lubricin protects cartilage surfaces and inhibits synovial cell overgrowth. J Clin Invest.

[35] Zhang X, Goncalves R, Mosser DM (2008). The isolation and characterization of murine macrophages. Curr Protoc Immunol.

[36] Kraus VB, Huebner JL, Fink C, King JB, Brown S (2002). Urea as a passive transport marker for arthritis biomarker studies. Arthritis Rheum.

[37] Elsaid KA, Zhang L, Shaman Z, Patel C, Schmidt TA (2015). The impact of early intra-articular administration of interleukin-1 receptor antagonist on lubricin metabolism and cartilage degeneration in an anterior cruciate ligament transection model. Osteoarthr Cartil.

[38] Orlowsky EW, Stabler TV, Montell E, Verges J, Kraus VB (2014). Monosodium urate crystal induced macrophage inflammation is attenuated by chondroitin sulphate: pre-clinical model for gout prophylaxis?. BMC Musculoskelet Disord.

[39] Zappone B, Ruths M, Greene GW, Jay GD, Israelachvili JN (2007). Adsorption, lubrication, and wear of lubricin on model surfaces: polymer brush-like behavior of a glycoprotein. Biophys J.

[40] Corfield AP (2015). Mucins: a biologically relevant glycan barrier in mucosal protection. Biochem Biophys Acta.

[41] Kosinska MK, Ludwig TE, Liebisch G, Zhang R, Siebert HC (2015). Articular joint lubricants during osteoarthritis and rheumatoid arthritis display altered levels and molecular species. PLoS One.

[42] Jay GD, Waller KA (2014). The biology of lubricin: near frictionless joint motion. Matrix Biol.

[43] Jay GD, Torres JR, Rhee DK, Helminen HJ, Hytinnen MM (2007). Association between friction and wear in diarthrodial joint lacking lubricin. Arthritis Rheum.

[44] Waller KA, Zhang LX, Elsaid KA, Fleming BC, Warman ML (2013). Role of lubricin and boundary lubrication in the prevention of chondrocyte apoptosis. Proc Natl Acad Sci U S A.

[45] Hill A, Walker KA, Allen JM, Smits P, Zhang LX (2015). Lubricin restoration in a mouse model of congenital deficiency. Arthritis Rheumatol.

[46] Waller KA, Zhang LX, Jay GD (2017). Friction-induced mitochondrial dysregulation contributes to joint deterioration in *Prg4*^*−/−*^ mice. Int J Mol Sci.

[47] Ponta H, Sherman L, Herrlich PA (2003). CD44: from adhesion molecules to signaling regulators. Nat Rev Mol Cell Biol.

[48] Fu Q, Wei Z, Xiao P, Chen Y, Liu X (2017). CD44 enhances macrophage phagocytosis and plays a protective role in Streptococcus equi subsp. zooepidemicus infection. Vet Microbiol.

[49] Vachon E, Martin R, Kwok V, Cherepanov V, Chow CW (2007). CD44-mediated phagocytosis induces inside-out activation of complement receptor-3 in murine macrophages. Blood.

[50] Kwana H, Karaki H, Higashi M, Miyazaki M, Hiberg F (2008). CD44 suppresses TLR-mediated inflammation. J Immunol.

[51] Liang J, Jiang D, Griffith J, Yu S, Fan J (2007). CD44 is a negative regulator of acute pulmonary inflammation and lipopolysaccharide-TLR signaling in mouse macrophages. J Immunol.

[52] Estrella RP, Whitelock JM, Packer NH, Karlesson NG (2010). The glycosylation of human synovial lubricin: implication for its role in inflammation. Biochem J.

[53] Jin C, Ekwall AK, Bylund J, Björkman L, Estrella RP (2012). Human synovial lubricin expresses sialyl Lewis x determinant and has L-selectin ligand activity. J Biol Chem.

[54] Jones AR, Gleghorn JP, Hughes CE, Fitz LJ, Zollner R (2007). Binding and localization of recombinant lubricin to articular cartilage surfaces. J Orthop Res.

[55] Torres R, McDonald L, Croll SD, Reinhardt J, Dore A (2009). Hyperalgesia, synovitis and multiple biomarkers of inflammation are suppressed by interleukin 1 inhibition in a novel animal model of gouty arthritis. Ann Rheum Dis.

[56] Edwards NL, So A (2014). Emerging therapies for gout. Rheum Dis Clin N Am.

[57] Ottaviani S, Molto A, Ea HK, Neuveu S, Gill G (2013). Efficacy of Anakinra in gouty arthritis: a retrospective study of 40 cases. Arthritis Res Ther.

[58] Pulli B, Ali M, Forghani R, Schob S, Hsieh KC (2013). Measuring myeloperoxidase activity in biological samples. PLoS One.

[59] Hampton MB, Kettle AJ, Winterbourn CC (1998). Inside the neutrophil phagosome: oxidants, myeloperoxidase, and bacterial killing. Blood.

[60] Coderre TJ, Wall PD (1987). Ankle joint urate arthritis (AJUA) in rats: an alternative animal model of arthritis to that produced by Freund’s adjuvant. Pain.

[61] Lee HS, Lee CH, Tsai HC, Salter DM (2009). Inhibition of cyclooxygenase 2 expression by diallyl sulfide on join inflammation induced by urate crystal and IL-1β. Osteoarthr Cartil.

[62] Silva CR, Oliveira SM, Hoffmeister C, Funck V, Guerra GP (2016). The role of kinin B1 receptor and the effect of angiotensin I-converting enzyme inhibition on acute gout attacks in rodents. Ann Rheum Dis.

[63] Jay GD, Elsaid KA, Kelly KA, Anderson SC, Zhang L (2012). Prevention of cartilage degeneration and gait asymmetry by lubricin tribosupplementation in the rat following anterior cruciate ligament transection. Arthritis Rheum.

[64] Jay GD, Fleming BC, Watkins BA, McHugh KA, Anderson SC (2010). Prevention of cartilage degeneration and restoration of chondroprotection by lubricin tribosupplementation in the rat following anterior cruciate ligament transection. Arthritis Rheum.

[65] Cui Z, Xu C, Li X, Song J, Yu B (2015). Treatment with recombinant lubricin attenuates osteoarthritis by positive feedback loop between articular cartilage and subchondral bone in ovariectomized rats. Bone.

[66] Teeple E, Elsaid KA, Jay GD, Zhang L, Badger GJ (2011). Effects of supplemental intra-articular lubricin and hyaluronic acid on the progression of posttraumatic arthritis in the anterior cruciate ligament-deficient rat knee. Am J Sports Med.

[67] Goldberg EL, Asher JL, Malony RD, Shaw AC, Zeiss CJ (2017). β-hydroxybutyrate deactivates neutrophil NLRP3 inflammasome to relieve gout flares. Cell Rep.

